# A first case report of using chimeric antigen receptor T-cell immunotherapy to treat high-risk smoldering multiple myeloma

**DOI:** 10.2478/jtim-2023-0097

**Published:** 2023-09-02

**Authors:** Yang Liu, Wenbing Duan, Xiaojun Huang, Jin Lu

**Affiliations:** Peking University People’s Hospital, Peking University Institute of Hematology, National Clinical Research Center for Hematologic Disease, Beijing Key Laboratory of Hematopoietic Stem Cell Transplantation, Peking University, Beijing 100044, China; Innovative Center of Hematology, Soochow University, Suzhou 215021, Jiangsu Province, China

Smoldering multiple myeloma (SMM) is now considered a heterogeneous set of diseases with different tendencies to progress to active myeloma. Several trials aimed at curing multiple myeloma (MM) in the smoldering stage through intensive treatment have reported effective control of active myeloma.^[1]^ At the same time, Chimeric antigen receptor (CAR) T-cell immunotherapy is currently being intensively tested for treating relapsed and refractory myeloma.^[2]^ Here, we report a patient with smoldering myeloma who elected to be treated with CAR T-cell therapy and has now achieved minimal residual disease (MRD) negativity.

On June 2017, a 30-year-old man´s routine health examination revealed a white blood cell count (WBC) of 2.0 × 109/L, but with no fever, no cough or expectoration, no bone pain, no fatigue, no abdominal pain or diarrhea. The patient was then referred to the outpatient department of Peking University People’s Hospital, Beijing, China. Routine blood examination (July 4, 2017) revealed WBC 2.85 × 109/L, Hemoglobulin (Hb) 152 g/L, Platelet (PLT) 177 × 109/L. Potential underlying factors for leucopenia were all negative, i.e. the autoantibody spectrum, Cytomegalovirus (CMV) immunoglobulin M (IgM) and parvovirus B19 IgM, ferritin, folic acid, Vitamin B12. However, biochemical tests showed a total protein level of 87.2 g/L, albumin 45.0 g/L, creatinine 87 μmol/L, and blood calcium 2.34 mmol/L. The level of immunoglobulin G (IgG) was 25.7 g/L, so we ran serum protein electrophoresis and found M protein at 11.3 g/L, with serum immunofixation electrophoresis (IFE) indicating IgG λ positivity (Figure 1). Bone marrow (BM) analysis showed 5.5% of mature plasma cells. The immunophenotype of BM cells was that CD38+ CD138+ plasma cells accounted for 0.67% of nucleated cells, of which 75.11% expressed CD45, CD38, CD138, cλ, BCMA, CD56, CD200, CD9, CXCR4, while negative for CD7, CD10, CD19, CD34, CD117, CD33, cκ, CD28, CD22, CD20, and CD276 indicating that they were abnormal clonal plasma cells. Normal plasma cells accounted for only 17.19% of the CD38 + CD138+ BM cells. We then performed BM G banding and identified Chromosome 46, XY [13/20] and BM interphase fluorescence in situ hybridization (FISH) showed no abnormalities. Whole body PET-CT indicated no bone destruction. Based on the above findings, monoclonal gammopathy of undetermined significance (MGUS) was diagnosed. No treatment targeting the plasma cell dyscrasia was recommended. However, the patient was anxious about his disease and eager to be treated. On April 2018, repeated BM biopsies showed that plasma cells now accounted for 10% of cells and thus SMM could be diagnosed. CD138 magnetic bead sorting combined with FISH results showed t (4; 14). A second PET-CT scan remained negative. Entering a clinical trial of lenalidomide, ixazomib and dexamethasone for patients with high-risk smoldering multiple myeloma was suggested to the patient. However, due to a low white blood cell count, he still failed to meet the inclusion criteria although he received prednisone 30 mg daily for a week.

**Figure 1:**
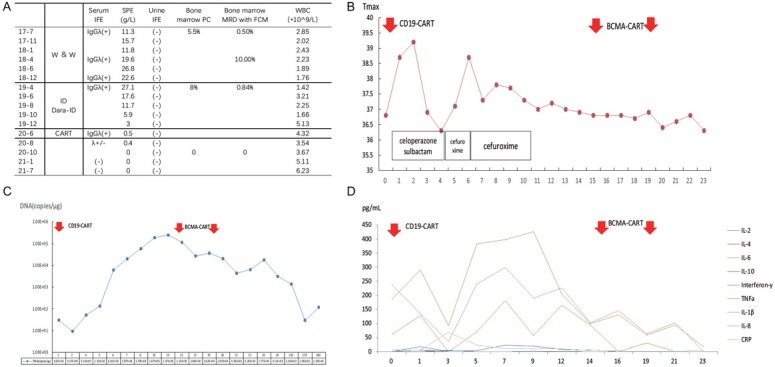
Patient with Smoldering Multiple Myeloma Treated with CD19 and BMCA Chimeric Antigen Receptor (CAR) T- Cell Immunotherapy. A. The laboratory changes during each line of therapy; B. The maximum temperature during CAR T-cell therapy; C. The peripheral CAR T-cell copy numbers /ug DNA changes during and after CAR T-cell infusions; D. The cytokine spectrum changes during CAR T-cell therapy. Abbreviation. IFE, immunofixation electrophoresis; SPE, serum protein electrophoresis; PC, plasma cell; MRD, minimal residual disease; FCM, flow cytometry; WBC, white blood cell; W & W, watch and wait; ID, ixazomib dexamethasone; Dara, daratumumab; IL, interleukin; TNF, tumor necrosis factor.

During the follow-up, the patient’s IgG and λ light chain increased while the white blood cell count decreased gradually, and M protein fluctuated around 15.7–27.1g/L. The patient continued to express a strong desire to receive treatment. Because M protein increased over time up to >20 g/L and he had a high-risk cytogenetic aberration, on April 26 2019, after thorough case discussion, we decided to offer the patient treatment. We detailed the benefits of various current treatments and fully communicated with the patient. As he had a strong desire to embark on a curative treatment and the most likely such therapeutic treatment was Chimeric antigen receptor (CAR) T-cell immunotherapy, it was planned to exploit this approach. Before CAR T-cell therapy, we planned to reduce the tumor load in order to minimize severe of cytokine releasing syndrome (CRS). Considering the adverse effects of lenalidomide and daratumumab on hematopoietic stem cell harvesting, we first administered the ID regimen (ixazomib 4 mg on day 1, 8, and 15, plus dexamethasone 40 mg once a week). The stem cells were collected on October 2019. Mononuclear cells (MNC) and CD34+ cells were 21.37 × 108/kg and 5.18 × 106/kg, respectively. After applying the ID regimen, partial remission (PR) was achieved. In order to maximally reduce the tumor load, after collection, daratumumab (16 mg/kg) and ID were given. A very good partial response (VGPR) was achieved. Due to the outbreak of coronavirus disease 2019 (COVID-19), the CAR T-cell therapy scheduled had to be delayed, during which the patient’s disease was stable.

On June 17 2020, the patient received lymphodepletion chemotherapy with the FC regimen (cyclophosphamide 300 mg/m^2^ × 3 days and fludarabine 25 mg/m^2^ × 3 days). Two days after this chemotherapy, autologous CD19 CAR T cells provided by the Unicar-Therapy Bio-Medicine Technology Co. (Shanghai, China) at a total dose of 5 × 10^6^ cells per kilogram were infused on June 22nd, 2020. After infusion, CRS grade 1 occurred. We treated the patient with antibiotics including celoperazone sulbactam, cefuroxime, piperacillin/tazobactam because infection could not be definitively excluded. After two weeks, we gave the patient two doses of anti-BCMA CAR T cells provided by the same company with a total dose of 1 × 10^7^ cells per kilogram over 3 days, with no CRS. After CAR T-cell therapy, no MRD was detected. In March 2022, MM remained MRD-negative. As of Sep 5^th^ 2022, the patient felt well and was continuing follow-up.

## Discussion

To the best of our knowledge, this is the first case of CAR T-cell therapy for high-risk smoldering myeloma. Anti-BCMA CAR T-cell therapy has shown impressive anti-myeloma activities (81–100%) in certain preclinical and/or clinical investigations.^[3]^ Because of its high efficiency, some investigators are trying to move this type of treatment forward, such as in the consolidation of high-risk MM. The theoretical basis for using CAR T-cell therapy in the early stage of MM is that the tumor burden is closely related to the adverse reactions of CAR T-cell therapy. The CRS reaction is associated with the interaction of myeloma cells and CAR T cells. As thus, like the KRd (carfilzomib-lenalidomide-dexamethasone) study in high-risk MM,^[4]^ if we can nip MM in the bud, CAR T-cell therapy is a feasible therapeutic option. For the present SMM patient with a tendency to progress, after induction therapy to reduce the tumor load, we accomplished lymphocytapheresis and succeeded in preparing the CD19- and BCMA-targeted chimeric antigen receptor T cells. Adverse effects were mild with only grade 1 CRS. Although the follow-up time was short, the response was already MRD negativity.

Because the efficacy of anti-BCMA therapy has been demonstrated and there are reports that a small subgroup of less-differentiated myeloma clones expresses CD19, a combination of humanized anti-CD19 and anti-BCMA CAR T-cell infusions seemed reasonable to us. Previously, sequential anti-CD19 and anti-BCMA CAR T-cell therapy had shown efficacy in relapsed and refractory myeloma with 95% overall and 57% complete responses. Grade 2 CRS was noted in 33% of recipients and even grade 3 in one case.^[5]^ Our case only had grade 1 CRS and was easily manageable, which may have been related to our strategy of reducing the tumor burden before initiating CAR T-cell therapy.

One other issue that needs pointing out regarding this patient is the relationship between the leucopenia and plasma cell disease. Here, leucopenia was possibly due to plasma cell dyscrasia because no evidence of other underlying causes was found, and after plasma cell treatment, the white blood cell count had returned to normal. For quite some time, “Slim-CRAB” has been deemed to be the prime indicator for myeloma treatment. Findings from our case reported here suggested that there may be some relationship between white blood cell counts and plasma cell dyscrasia. The connection between the two kinds of cells’ abnormalities is consistent with observations that chronic neutrophil leukemia tends to coexist with plasma cell disease.^[6]^ For this case, after treatment, the WBC count recovered completely.

## Conclusion

This is the first report of treatment by sequential anti-CD19 and then anti-BCMA CAR T-cell immunotherapy in a patient with smoldering multiple myeloma. Our findings suggest that anti-BCMA and CD 19 CAR T-cell therapy may be a feasible therapeutic option for patients with high-risk SMM. Early intervention with CAR T-cell therapy at the smoldering stage of MM when the immune microenvironment is still relatively normal and clonal plasma cells do not yet have complex mutations may possibly offer an exceptional opportunity to cure multiple myeloma.
